# Alamandine attenuates stress-induced inflammation, oxidative stress, and intestinal histopathological alterations in a chronic unpredictable mild stress model of depression

**DOI:** 10.1007/s00210-026-05612-2

**Published:** 2026-07-03

**Authors:** Aysu Kilic, Betul Esra Ipek, Yasin Ali Cimen, Mert Yilmaz, Fatma Bedia Karakaya Cimen, Emine Rumeysa Hekimoglu, Huri Demirci, Savas Ustunova

**Affiliations:** 1 Department of Physiology, School of Medicine, Bezmialem Vakif University, Istanbul, Türkiye; 2 Department of Physiology, School of Medicine, Yalova University, Istanbul, Türkiye; 3 Department of Histology and Embryology, School of Medicine, Yalova University, Istanbul, Türkiye; 4 Department of Histology and Embryology, School of Medicine, Bezmialem Vakif University, Istanbul, Türkiye; 5 Department of Medical Biochemistry, School of Medicine, Biruni University, Istanbul, Türkiye

**Keywords:** Alamandine, Chronic unpredictable mild stress, Depression, Gut, Inflammation, Renin-angiotensin system

## Abstract

Depression is increasingly recognized as a systemic disorder associated with chronic inflammation, oxidative stress, and disturbances in the gut–brain axis. This study aimed to investigate whether alamandine modulates chronic stress–induced alterations in the intestine and liver by assessing histopathological changes, inflammatory responses, and oxidative stress parameters in a chronic unpredictable mild stress (CUMS) model. Male rats were exposed to CUMS for 35 days, and the effects of Alamandine and the Mas receptor antagonist A779 were evaluated. Serum inflammatory cytokines (TNF-α, IL-6), liver cytokines (IL-6, IL-1β), oxidative stress markers including superoxide dismutase (SOD), catalase (CAT), and malondialdehyde (MDA), as well as biochemical parameters (AST, ALT, total protein) were analyzed. Histopathological examination of intestinal and colonic tissues was also performed. CUMS significantly increased serum cortisol and pro-inflammatory cytokines, indicating systemic inflammation, while elevated IL-6 and IL-1β levels in liver tissue suggested a localized inflammatory response. Oxidative stress findings showed impaired antioxidant defense and increased lipid peroxidation. Despite these changes, AST, ALT, and total protein levels remained unchanged, indicating no overt hepatic injury. Histologically, CUMS caused intestinal villus degeneration, inflammatory infiltration, and reduced goblet cell numbers, whereas the colon exhibited milder epithelial alterations. Alamandine treatment attenuated inflammatory and oxidative responses and improved intestinal mucosal integrity, with partial restoration of goblet cells. These findings suggest that alamandine attenuates chronic stress–induced inflammatory, oxidative, and histopathological alterations in intestinal and hepatic tissues. The results further support a potential protective role of the RAS pathway in peripheral tissue injury associated with chronic stress.

**Graphical Abstract:**

Chronic stress induces inflammation, oxidative stress, and intestinal mucosal damage. Alamandine attenuates these alterations and improves intestinal histopathological changes through modulation of the protective RAS pathway.

**Figure:**
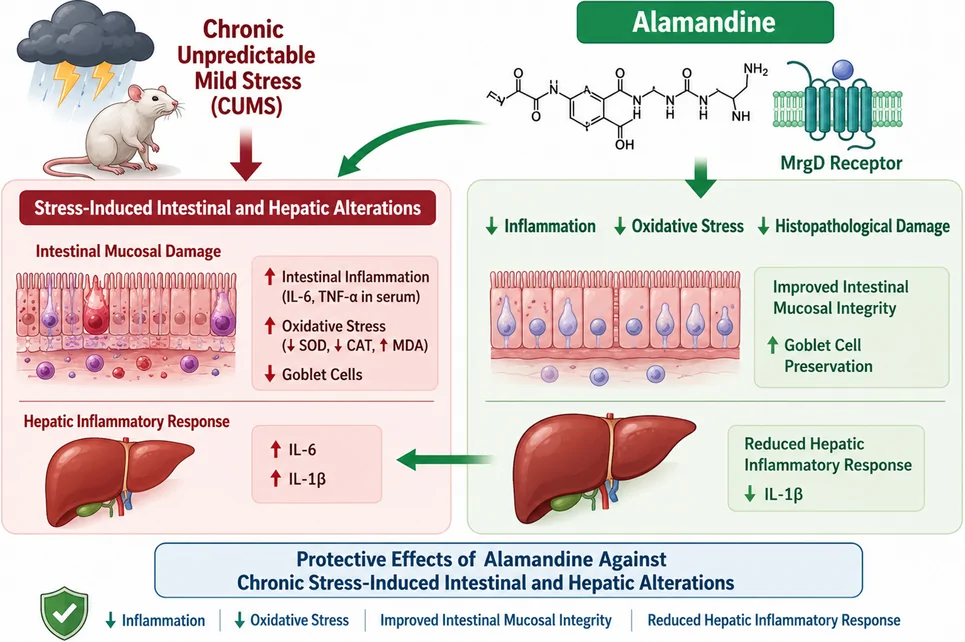

## Introduction

Depression is one of the most prevalent psychiatric disorders worldwide and is increasingly recognized as a systemic disorder associated with chronic low-grade inflammation, oxidative stress, and neuroendocrine dysfunction (Beckett and Lawson 2026; Hassamal 2023). Sustained activation of the hypothalamic–pituitary–adrenal (HPA) axis during chronic stress results in elevated glucocorticoid levels, increased production of reactive oxygen species (ROS), and enhanced release of pro-inflammatory cytokines, including TNF-α, IL-1β, and IL-6 (Bhatt et al. 2020; Dowlati et al. 2010; Ng et al. 2018; P and Vellapandian 2024). These alterations extend beyond the central nervous system and contribute to dysfunction in peripheral organs, particularly the gastrointestinal tract and liver. Clinical and experimental evidence indicates a close relationship between depression and gastrointestinal or hepatic disorders, suggesting shared pathophysiological mechanisms linking the brain and peripheral organs (Kronsten et al. 2022; Menon et al. 2022).

The gut–brain axis is a bidirectional communication network involving neural, endocrine, immune, and metabolic pathways. Chronic stress disrupts this network by impairing intestinal barrier integrity, increasing intestinal permeability, and promoting inflammatory signaling (Foster et al. 2017; Kelly et al. 2015; Söderholm and Perdue 2001). The resulting translocation of microbial products and inflammatory mediators into the circulation may directly affect the liver through the portal system, contributing to the development of a functional gut–liver axis during chronic stress.

The renin–angiotensin system (RAS), traditionally known for its role in cardiovascular regulation, has recently emerged as an important mediator of both depression and peripheral inflammation (Ranjbar et al. 2019). The classical ACE/Angiotensin II/AT1R axis promotes oxidative stress, inflammation, and tissue injury, whereas the counter-regulatory ACE2/Angiotensin-(1–7)/Mas axis exerts anti-inflammatory, antioxidant, and tissue-protective effects (Miyazaki et al. 2006; Santos et al. 2018). Accumulating evidence indicates that dysregulation of the RAS contributes to the pathophysiology of chronic stress and depression. Experimental studies have demonstrated that chronic stress enhances activation of the classical RAS pathway, contributing to neuroinflammation, HPA axis dysregulation, and depressive-like behaviors. In contrast, stimulation of the protective ACE2/Ang-(1–7)/Mas axis attenuates these pathological processes and improves behavioral outcomes (Campagnole-Santos et al. 2025; Gong and Deng 2022; Sukumaran et al. 2012).

Alamandine, a recently identified component of the protective RAS axis, is generated from angiotensin A via ACE2 or from Angiotensin-(1–7) through enzymatic decarboxylation and exerts its effects primarily through the Mas-related G-protein–coupled receptor D (MrgD) (Hrenak et al. 2016; Lautner et al. 2013; Villela et al. 2014). Previous studies have demonstrated that alamandine possesses anti-inflammatory, antioxidant, antifibrotic, and tissue-protective properties, partly through suppression of NF-κB signaling and reduction of oxidative damage (de Carvalho Santuchi et al. 2019). Moreover, activation of the alamandine/MrgD axis has been associated with antidepressant-like effects (Almeida-Santos et al. 2021). In our previous study, alamandine improved spatial memory and reduced anxiety-like behavior in chronically stressed rats by modulating neuroinflammation and brain-derived neurotrophic factor (BDNF) levels in the hippocampus and prefrontal cortex (Kilic et al. 2025). However, its potential effects on peripheral inflammatory responses and gut–liver axis dysfunction under chronic stress conditions remain unclear.

To further investigate the contribution of the protective RAS pathway, the selective Mas receptor antagonist A-779 has been widely used as a pharmacological tool to block Angiotensin-(1–7)/Mas receptor signaling. By antagonizing Mas receptor activation, A-779 abolishes many of the anti-inflammatory, antioxidant, and tissue-protective effects attributed to the ACE2/Ang-(1–7)/Mas axis, thereby allowing the functional characterization of protective RAS-mediated mechanisms (Santos et al. 2018). Therefore, A-779 was included in the present study to evaluate the potential involvement of Mas-related signaling in the observed effects of alamandine under chronic stress conditions. To investigate the involvement of protective RAS signaling, the selective Mas receptor antagonist A-779 was used as a pharmacological tool to block ACE2/Ang-(1–7)/Mas receptor activity (Santos et al. 2018). Therefore, the present study aimed to evaluate the protective effects of alamandine against chronic stress–induced gut–liver injury by histopathological assessment of intestinal and hepatic tissues and by evaluating oxidative stress and inflammatory parameters in a chronic unpredictable mild stress model.

## Materials and methods

### Ethical approval and animals

Thirty-five male Sprague–Dawley rats (350–400 g) were obtained from the Bezmialem Vakıf University Experimental Animal Center. Only male rats were used to avoid variability associated with the estrous cycle and hormonal fluctuations that may influence inflammatory and stress-related responses, in accordance with the literatüre (Markov and Novosadova 2022). Animals were housed under standard laboratory conditions (12 h light/dark cycle, 22 ± 2 °C, 60% humidity) with ad libitum access to food and water. All procedures complied with national and institutional guidelines for laboratory animal welfare (ARRIVE guidelines; Kilkenny et al., 2010) and were approved by the Bezmialem Vakıf University Animal Experiments Local Ethics Committee (06.02.2023-E.94371).

### Experimental groups and treatments

Animals were randomly assigned to five groups (*n* = 7 per group):

- **Control (C)** – standard housing, saline injection
- **Chronic Unpredictable Mild Stress (CUMS)**
- **CUMS + Alamandine (CUMS + Ala)**
- **CUMS + A-779 (CUMS + A779)**
- **CUMS + Alamandine + A-779 (CUMS + Ala + A779)**

Alamandine (Sigma-Aldrich, SML1374) and A-779 (Sigma-Aldrich, 23,396) were dissolved in saline. Alamandine (50 µg/kg, ip). The alamandine dose was selected based on the effective dose reported by Yang et al. and converted to the rat equivalent dose according to Reagan-Shaw et al. (Reagan-Shaw et al. 2008; Yang et al. 2020) and A-779 (300 µg/kg, ip) (Yuan et al. 2013) were administered daily for 21 days during the CUMS procedure. Control animals received equal volumes of saline.

### Chronic unpredictable mild stress protocol

Animals were exposed to randomly scheduled stressors for 35 days to model depression-related chronic stress (Sequeira-Cordero et al. 2019; Willner 2005). To maintain unpredictability, the same stressor was not repeated on three consecutive days. Each day, rats were subjected to a single stressor such as restraint, wet bedding, cage tilting, food/water deprivation, cold exposure, or social isolation, as previously described (Strekalova et al. 2022). All stressors were initiated at 09:00 a.m. and applied according to their designated durations throughout the CUMS protocol (Table 1).

**Table 1:** Schedule of stressor application

Stressor	Duration	Application Schedule (Days)
Hot plate (45°C)	5 min	1, 24, 32
Cold plate (5°C)	5 min	9, 27, 35
Restraint	4 h	2, 16, 28
Wet bedding	24 h	10, 19, 34
Food deprivation	24 h	5, 12, 22
Water deprivation	24 h	13, 21, 33
Social isolation	24 h	4,17, 26
Cage tilting	24 h	3, 14, 20, 25
Crowded housing	24 h	8, 18, 30
Reversal of light/dark cycle	24 h	11, 23, 29
Tail clamping	1 min	7, 15, 31

In our previous study using the same experimental design and animal cohort, the CUMS model was behaviorally validated by a significant reduction in sucrose preference and an increase in anxiety-like behaviors across multiple behavioral paradigms (Kilic et al. 2025). The present study was conducted under a separate ethical approval and utilized tissues collected from these animals for molecular and histopathological analyses. In accordance with the 3R principles, particularly the principle of Reduction, behavioral validation procedures were not repeated in the current study.

### Sample collection

At the end of the experimental period, rats were anesthetized with ketamine (100 mg/kg) and xylazine (10 mg/kg) and decapitated. Blood, liver, small intestine, and colon tissues were collected. Liver samples were snap-frozen in liquid nitrogen and stored at − 80 °C for biochemical analyses. Intestinal tissues were fixed in 10% neutral buffered formalin for histological evaluation. Serum samples were obtained by centrifugation at 3,000 × g for 10 min and stored at − 80 °C.

### Biochemical analyses

Liver tissues were homogenized in phosphate-buffered saline (pH 7.4) using a bead homogenizer (Retsch MM400). After centrifugation (15,000 × g, 20 min, 4 °C), supernatants were collected. Total protein levels were determined using the Bradford method (Kruger 1994).

The levels of inflammatory cytokines, including tumor necrosis factor-alpha (TNF-α) (Bioassay Technology Laboratory, Shanghai, China, No: E0764Ra), interleukin-1 beta (IL-1β) (Bioassay Technology Laboratory, Shanghai, China, No: E0119Ra), and interleukin-6 (IL-6) (Bioassay Technology Laboratory, Shanghai, China, No: E0119Ra) E0135Ra), as well as oxidative stress and antioxidant parameters, including malondialdehyde (MDA) (Bioassay Technology Laboratory, Shanghai, China, No: E0156Ra), superoxide dismutase (SOD) (SunRed Shanghai, China, No: 201–11–0169), and catalase (CAT) (Bioassay Technology Laboratory, Shanghai, China, No: E0869Ra), were determined using enzyme-linked immunosorbent assay (ELISA) kits according to the manufacturers’ protocols. Briefly, the standards and samples were pipetted into microplates coated with a monoclonal antibody and incubated. Biotin was added to all wells, and streptavidin-HRP was added to induce binding. After incubation, 4 washes were performed to remove unbound reagents. After adding chromogen solutions A and B, a stop solution was added, and the resulting optical density was measured at 450 nm using a plate reader (Thermo Scientific, Varioskan™ LUX multimode microplate reader, USA).

Serum aspartate aminotransferase (AST), alanine aminotransferase (ALT), lipase, and total protein (TP) levels in serum samples were measured with a semiautomatic biochemical analyzer (IDEXX Vettest, IDEXX Laboratories, Inc., USA). Detection ranges for the ALT, AST, lipase, cholesterol, and TP kits.

### Histopathological evaluation

Intestinal and colon tissues were fixed in 10% buffered formalin, dehydrated through graded alcohols, embedded in paraffin, and sectioned at 4–5 µm thickness. Sections were stained with hematoxylin–eosin (H&E) and periodic acid–Schiff (PAS) and examined under a light microscope by two blinded histologists. For morphometric analysis, five intact villi encompassing the full crypt–villus axis were selected from each section, and villus height as well as goblet cell counts were measured at 10 × magnification. Histological assessment included evaluation of villus architecture, epithelial integrity, inflammatory cell infiltration in the lamina propria, goblet cell distribution, and regional differences between the small intestine and the colon. These parameters were chosen for their established relevance to stress-induced intestinal barrier dysfunction and mast cell–mediated mucosal injury.

### Statistical analysis

Data were analyzed using GraphPad Prism Software 8.0.1. Normality was assessed using the Shapiro–Wilk test. Parametric data were analyzed using one-way ANOVA followed by Tukey’s post hoc test, while non-parametric data were analyzed using the Kruskal–Wallis test with Dunn’s post hoc comparisons. Results are presented as mean ± SD, and *p* < 0.05 was considered statistically significant.

## Results

### Liver tissue cytokine levels

**Fig. 1:**
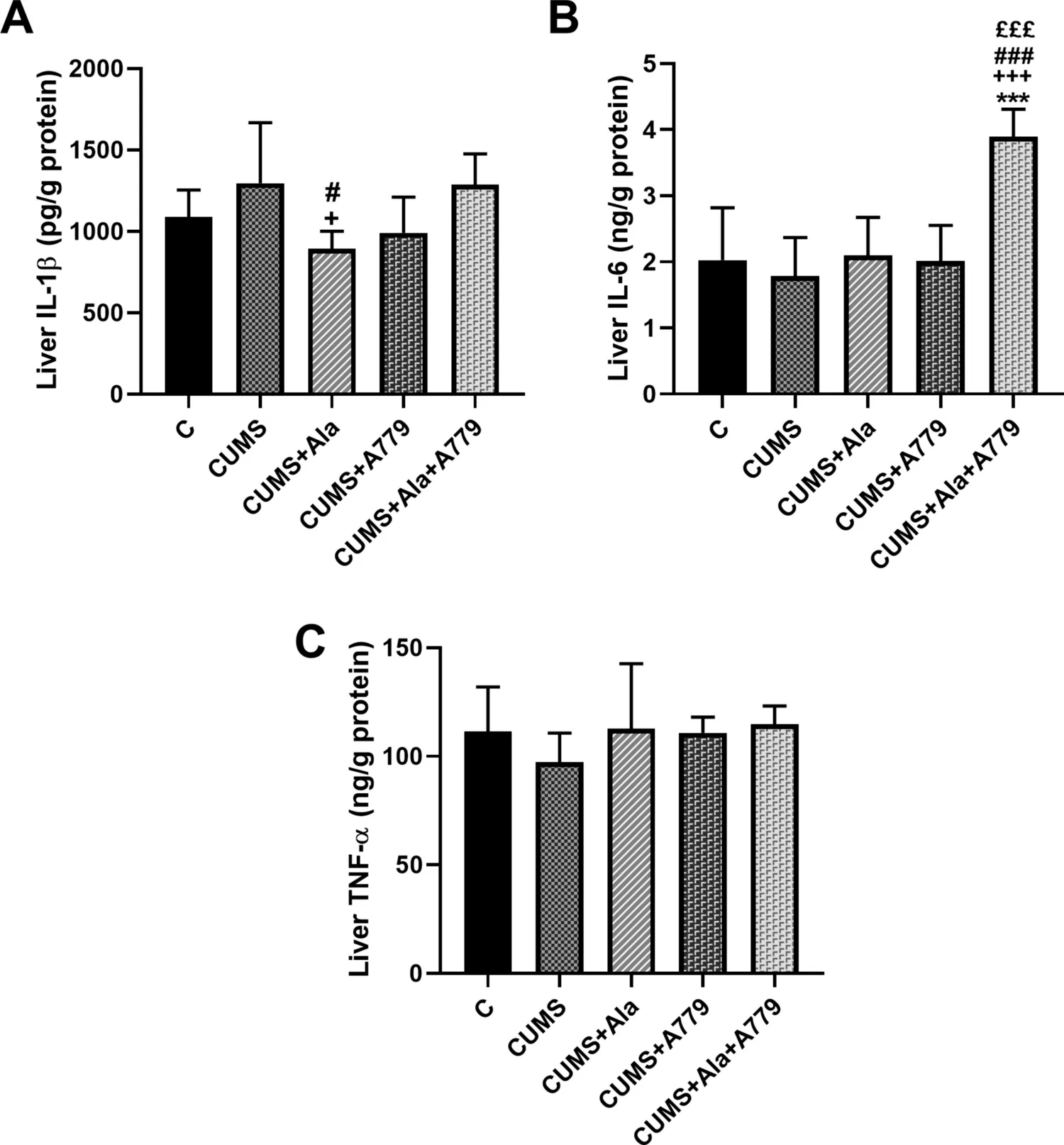
Biochemical results of liver tissue homogenates. Data were presented as mean ± SD. ***p < 0.001 statistical significance compared to the C group. ^**+**^*p* < 0.05, ^**+++**^*p* < 0.001 statistical significance compared to the CUMS group. ^**###**^*p* < 0.001 statistical significance compared to the CUMS + A779 group. ^**£**^*p* < 0.05, ^**£££**^*p* < 0.001, statistical significance compared to the CUMS + ALA group. TNF-α; Tumor Necrosis Factor-alpha, IL-1β; Interleukin-1 beta, IL-6; Interleukin-6

IL-1β levels showed a significant decrease in the CUMS + Ala group compared to the CUMS group (*p* < 0.05) and CUMS + A779 group (*p* < 0.05). No significant difference was observed between the other groups (Fig. 1A). IL-6 levels were significantly higher in the CUMS + Ala + A779 group compared to the control, CUMS, CUMS + Ala, and CUMS + A779 groups (*p* < 0.001) (Fig. 1B). No statistically significant differences were observed among the groups in TNF-α levels (Fig. 1C).

### Liver function parameters

**Fig. 2:**
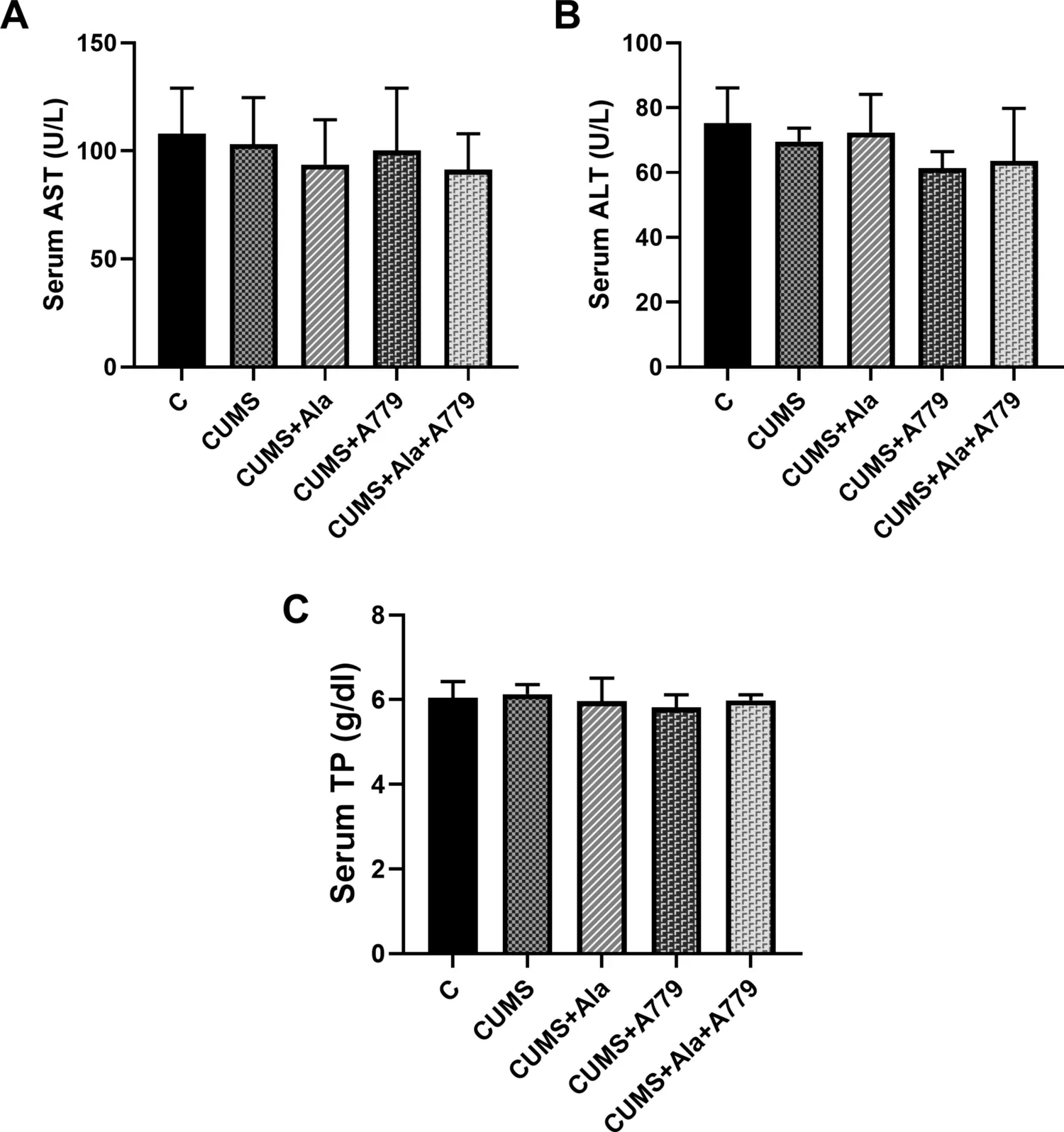
Biochemical results of serum samples. AST; Aspartate Aminotransferase, ALT; Alanine Aminotransferase, TP; Total Protein

No statistically significant differences were observed among the groups in terms of AST, ALT, or total protein levels (Fig. 2).

### Serum cytokine levels

**Fig. 3:**
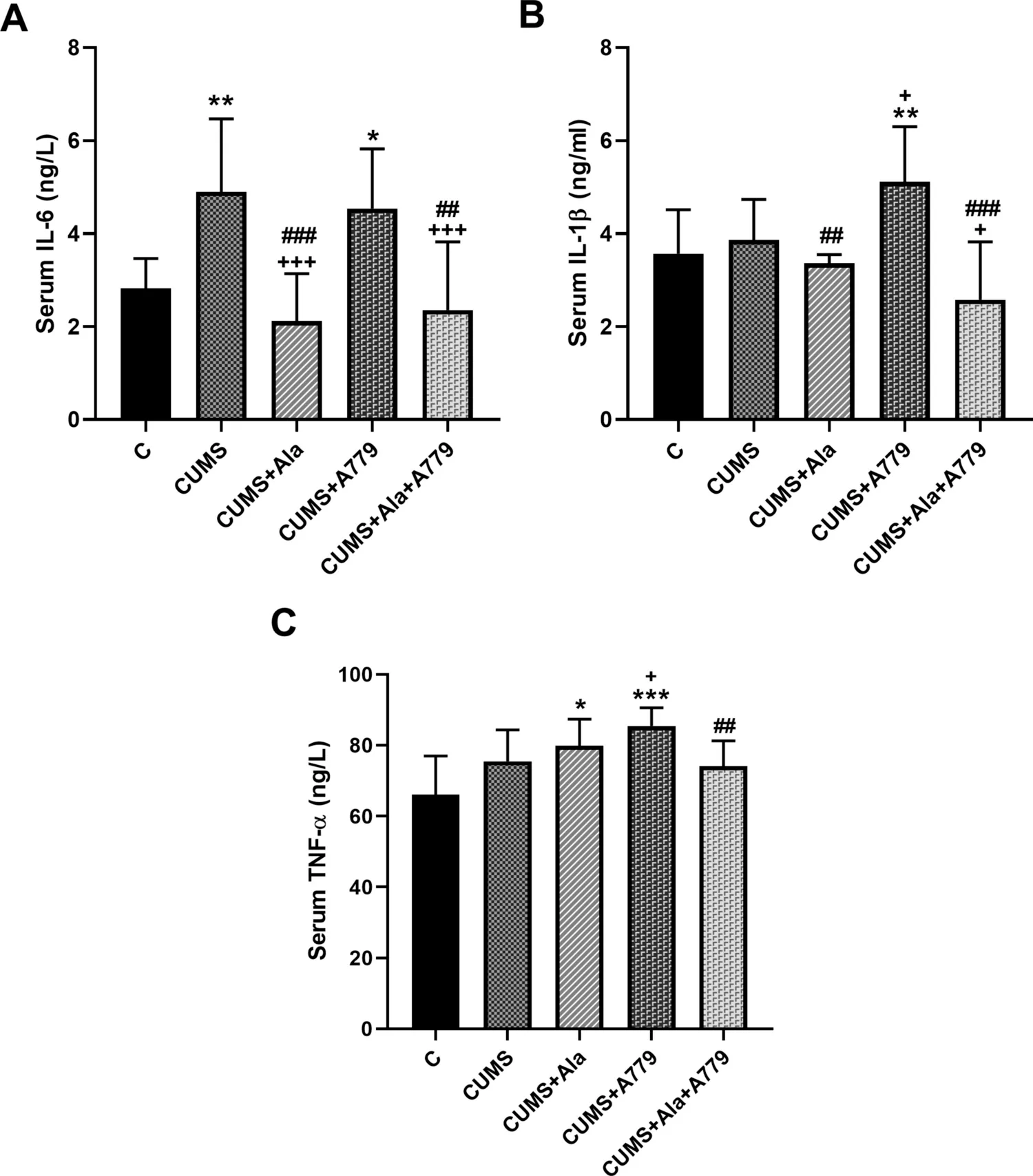
Biochemical results of serum samples. Data were presented as mean ± SD. **p < 0.01, ***p < 0.001 statistical significance compared to the C group. ^**+**^*p* < 0.05, ^**++**^*p* < 0.01, ^**+++**^*p* < 0.001 statistical significance compared to the CUMS group. ^**#**^*p* < 0.05, ^**##**^*p* < 0.01, ^**###**^*p* < 0.001 statistical significance compared to the CUMS + A779 group. TNF-α; Tumor Necrosis Factor-alpha, IL-1β; Interleukin-1 beta, IL-6; Interleukin-6

Serum IL-6 levels were significantly higher in the CUMS group compared to the control group (*p* < 0.01). Levels were significantly lower in the CUMS + Ala and CUMS + Ala + A779 groups compared to both the CUMS group (*p* < 0.001) and the CUMS + A779 group (*p* < 0.001, *p* < 0.01, respectively). The CUMS + A779 group showed higher IL-6 levels than in the controls (*p* < 0.05) (Fig. 3A).

The highest serum IL-1β levels were detected in the CUMS + A779 group, and this increase was statistically significant compared to the control (*p* < 0.01) and CUMS groups (*p* < 0.05). IL-1β levels were significantly lower in the CUMS + Ala (*p* < 0.01) and CUMS + Ala + A779 (*p* < 0.001) groups compared to the CUMS + A779 group. Lastly, levels of IL-1β were significantly lower in the CUMS + Ala + A779 group (*p* < 0.05) than in the CUMS group (Fig. 3B).

TNF-α levels were significantly elevated in the CUMS + Ala (*p* < 0.05) and CUMS + A779 (*p* < 0.001) groups compared to the control group. The highest levels of serum TNF-a were detected in the CUMS + A779 group, and this increment was statistically significant (*p* < 0.05) compared to the CUMS group. Levels were significantly reduced in the CUMS + Ala + A779 group compared to the CUMS + A779 group (*p* < 0.01) (Fig. 3C).

### Oxidative stress and cortisol parameters

**Fig. 4:**
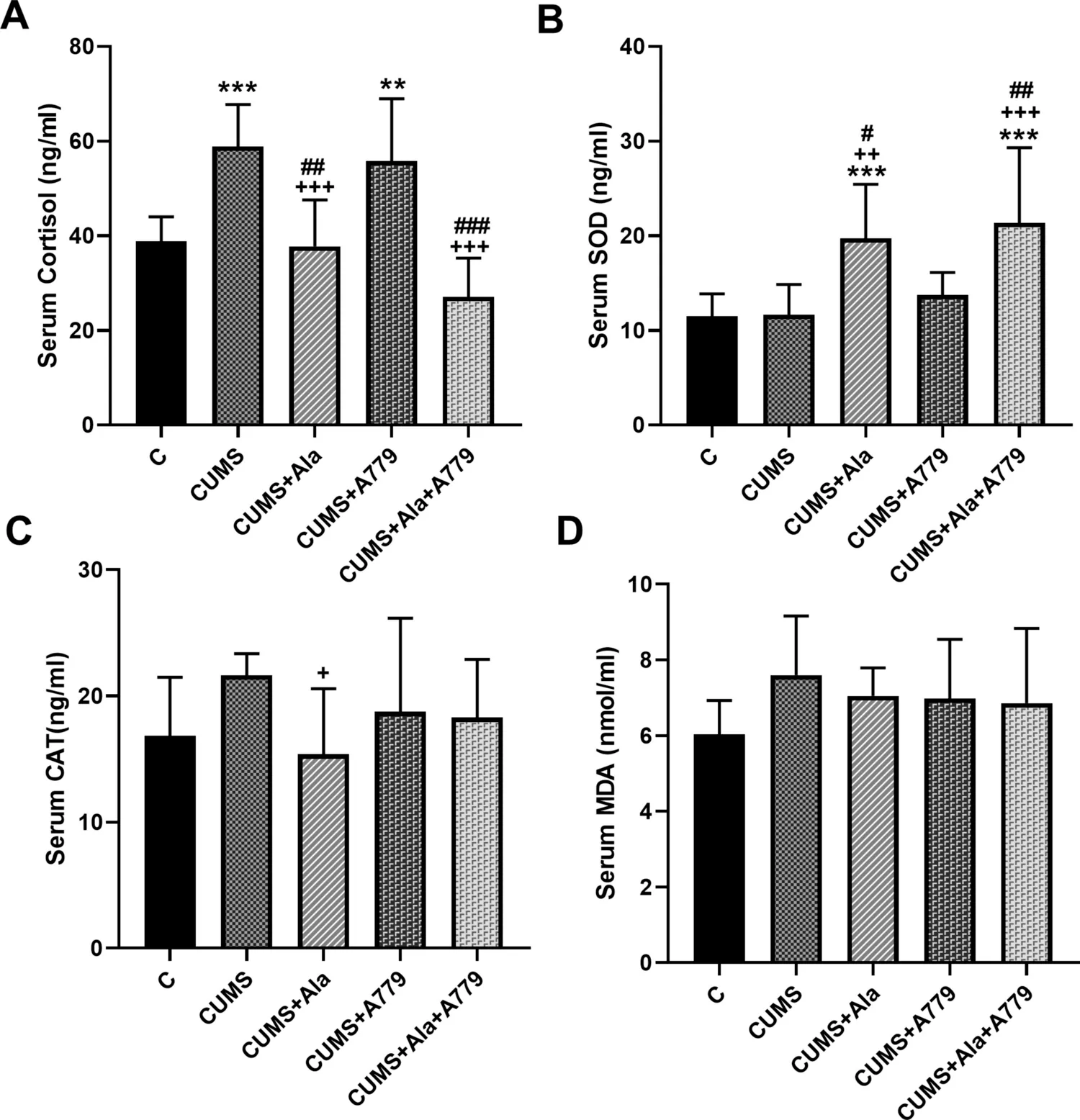
Biochemical results of serum samples. Data were presented as mean ± SD. *p < 0.05, **p < 0.01, ***p < 0.001 statistical significance compared to the C group. ^**+**^*p* < 0.05, ^**+++**^*p* < 0.001 statistical significance compared to the CUMS group. ^**##**^*p* < 0.01, ^**###**^*p* < 0.001 statistical significance compared to the CUMS + A779 group. SOD: Superoxide Dismutase, CAT: Catalase, MDA: Malondialdehyde

Serum cortisol levels were significantly higher in the CUMS (*p* < 0.001) and CUMS + A779 (*p* < 0.01) groups compared to controls. Levels were significantly lower in both CUMS + Ala and CUMS + Ala + A779 groups (p’s < 0.001) than in the CUMS group. Additionally, serum cortisol levels were significantly lower in both CUMS + Ala (*p* < 0.01) and CUMS + Ala + A779 (*p* < 0.001) groups than in the CUMS + A779 group (Fig. 4A).

SOD levels were significantly higher in the CUMS + Ala group compared to the control (*p* < 0.001) CUMS (*p* < 0.01 and CUMS + A779 (*p* < 0.05) groups. Levels of SOD were also higher in the CUMS + Ala + A779 group compared to the control (*p* < 0.001), CUMS (*p* < 0.001), and CUMS + A779 groups (*p* < 0.01) (Fig. 4B).

CAT levels were significantly lower in the CUMS + Ala group compared to the CUMS group (*p* < 0.05). No other significant differences were observed (Fig. 4C).

No statistically significant differences were observed in MDA levels among groups (Fig. 4D).

### Histopathological evaluation of intestinal tissue

#### Intestinal morphology

**Fig. 5:**
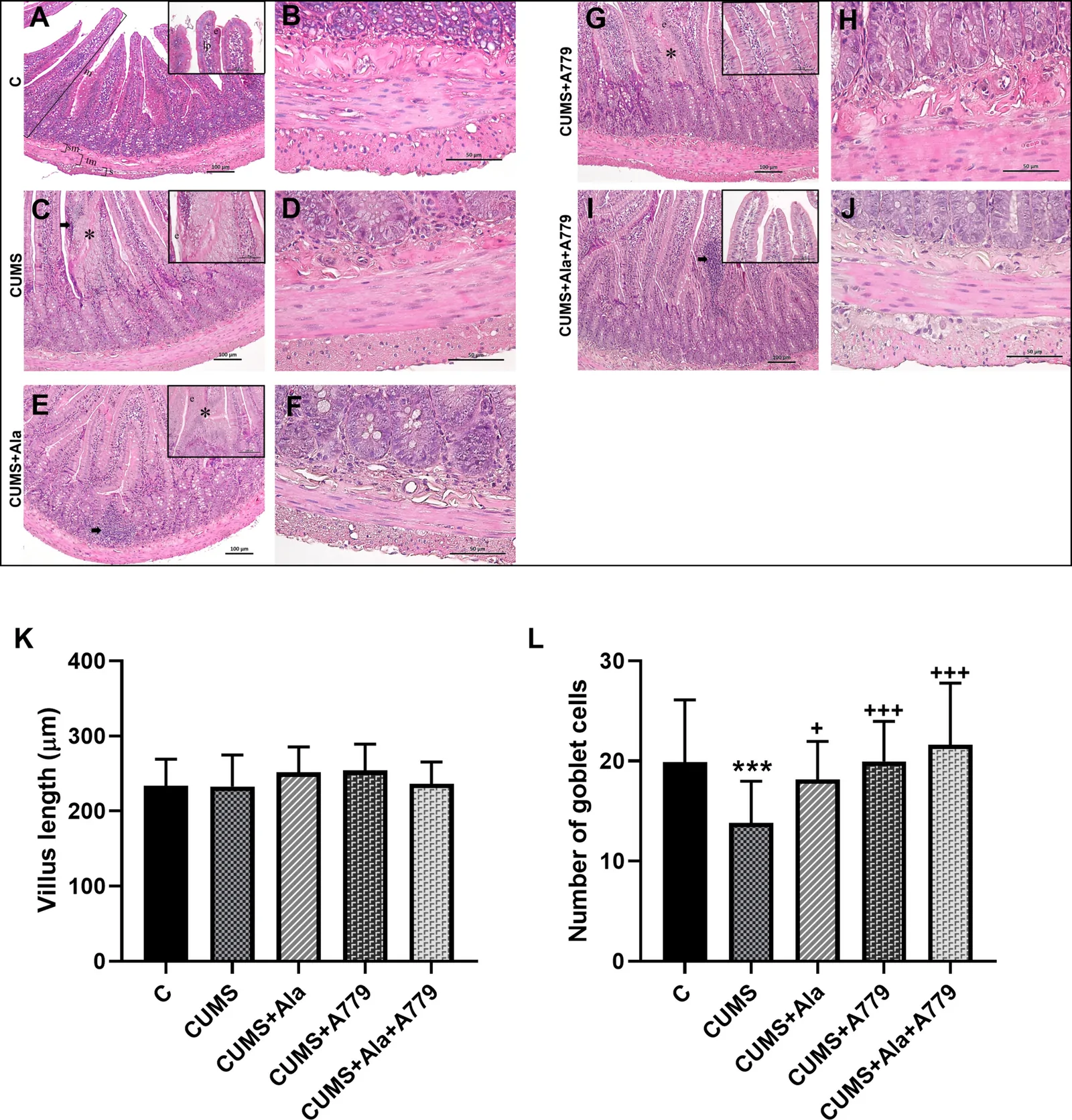
Light microscopic photographs of the rat small intestine (jejunum) of C (**A-B**), D (**C-D**), CUMS + ALA (E–F), CUMS + A779 (**G-H**), and CUMS + Ala + A779 (**I-J**) groups. (H&E X10, Inset: H&E X40). Tunica mucosa (m), submucosa (sm), tunica muscularis (tm), and serosa (s) layers. *Epitelial and villi degeneration of CUMS, CUMS + Ala and CUMS + A779 groups. An arrow represents inflammation. Statistical analysis of villus length (K) and number of goblet cells (L). Data were presented as mean ± SD. ***p < 0.001 statistical significance compared to the C group. ^+++^*p* < 0.001 statistical significance compared to the CUMS group

The control group displayed normal villus architecture and epithelial integrity (Fig. 5A-B). The CUMS group exhibited villus degeneration and inflammatory cell infiltration in the lamina propria (Fig. 5C-D). The CUMS + Ala group showed partial preservation of villus structure with reduced inflammatory infiltration (Fig. 5E-F). The CUMS + A779 group demonstrated persistent mucosal damage and inflammatory infiltration (Fig. 5G-H). The CUMS + Ala + A779 group exhibited improved mucosal architecture, comparable to that of the control group (Fig. 5I-J).

#### Villus length

No statistically significant differences in villus length were observed among groups (Fig. 5K).

#### Goblet cell density

Goblet cell numbers were significantly decreased in the CUMS group compared to controls (*p* < 0.001). Goblet cell counts were significantly increased in the CUMS + Ala, CUMS + A779, and CUMS + Ala + A779 groups compared to the CUMS group (*p* < 0.05–0.001) (Fig. 5L).

#### Histopathological evaluation of colon tissue

**Fig. 6:**
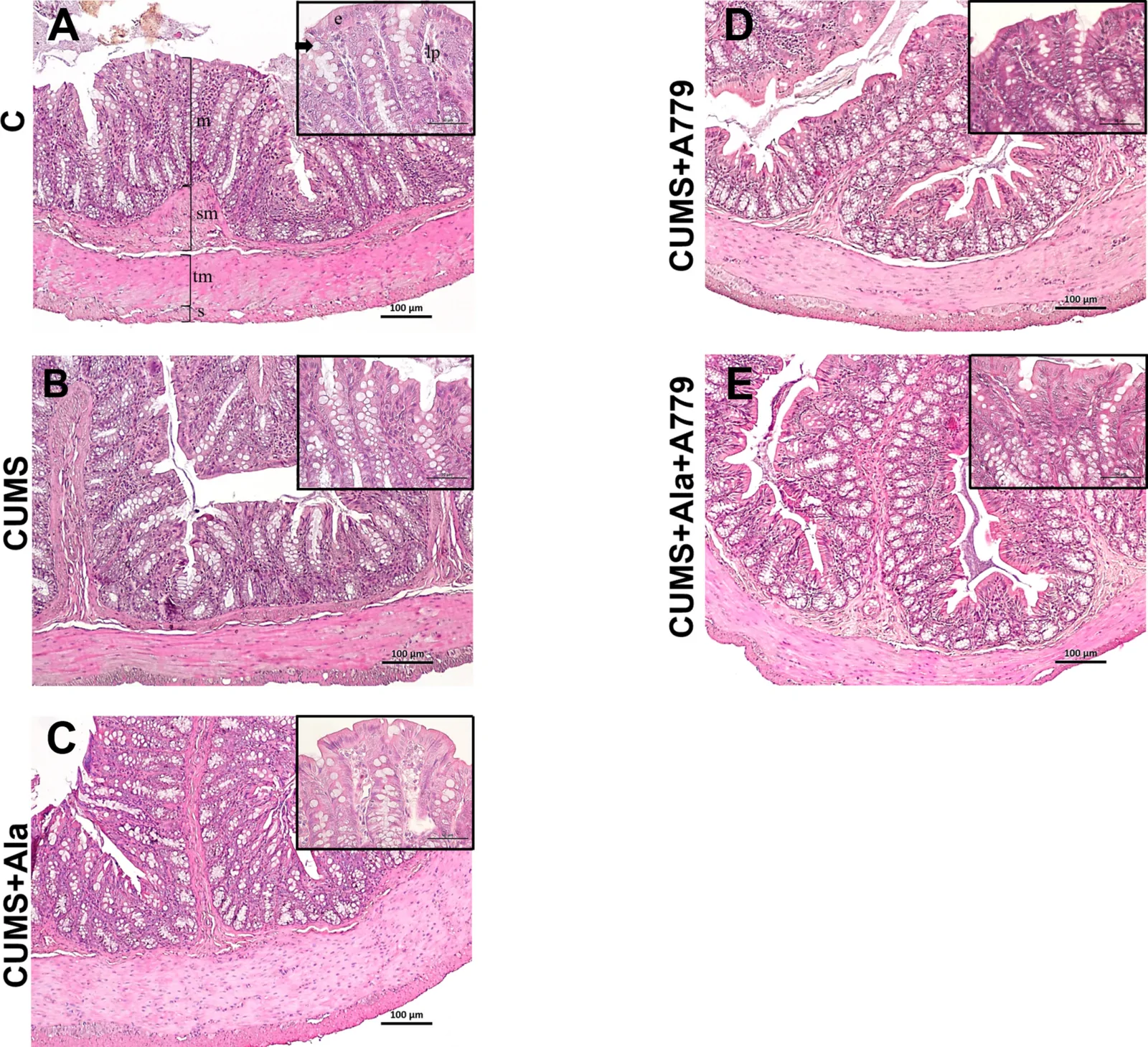
Representative light microphotographs of the rat colon. of C (**A**), CUMS (**B**), CUMS + Ala (**C**), CUMS + A779 (**D**), and CUMS + AlA + A779 (**E**) groups. (H&E X10, Inset: H&E X40). Tunica mucosa (m), submucosa (sm), tunica muscularis (tm), and serosa (s) layers

Histopathological evaluation of colon sections demonstrated that the C group preserved normal mucosal architecture, with intact surface epithelium, well-organized crypts, and physiological goblet cell distribution (Fig. 6A). In the CUMS group, mucosal injury was characterized by crypt architectural distortion, focal epithelial degeneration, inflammatory cell infiltration in the lamina propria, and mild mucosal thickening. Goblet cell density showed an irregular pattern compared to the control group (Fig. 6B). In the CUMS + Ala group, these alterations were markedly attenuated, with restoration of epithelial integrity, improved crypt organization, reduced inflammatory infiltration, and normalization of goblet cell distribution (Fig. 6C). Conversely, the CUMS + A779 group exhibited more pronounced mucosal damage, including evident crypt distortion, increased inflammatory infiltration, mucosal thickening, and goblet cell hyperplasia (Fig. 6D). In the CUMS + Ala + A779 group, partial histological recovery was observed, with improved crypt architecture, decreased inflammatory features, and a more balanced goblet cell profile compared to the D + A779 group (Fig. 6E).

## Discussion

Depression is increasingly recognized not merely as a localized neurological disorder but as a complex systemic pathology characterized by chronic low-grade inflammation. Prolonged exposure to stressors triggers an overactivation of the hypothalamic–pituitary–adrenal (HPA) axis. This neuroendocrine disruption leads to a persistent release of glucocorticoids and pro-inflammatory cytokines into circulation (Pariante and Lightman 2008). Our results align with these established models, as evidenced by the significant elevation of serum cortisol levels in the CUMS group. This elevation mirrors the sustained increase in corticosterone reported in the literature during 20 to 60 days of stress, representing an exhaustion phase in which the organism can no longer effectively counter the effects of stress (Willner 2017).

Although the present study did not directly evaluate brain tissue, the reduction in serum cortisol levels observed following alamandine treatment may suggest a beneficial effect on stress-related neuroendocrine responses. Since chronic stress is associated with HPA axis hyperactivity, neuroinflammation, and oxidative stress, the observed anti-inflammatory and antioxidant effects of alamandine may help protect against stress-induced alterations in the brain. Furthermore, our previous study demonstrated that alamandine reduced neuroinflammation and improved cognitive function in rats exposed to chronic stress (Kilic et al. 2025).

The systemic inflammatory profile observed in our study is consistent with the hallmark features of the CUMS model. The marked increase in serum IL-6 and TNF-α in our CUMS rats replicates findings from López-López et al. (2016), who identified an early and sustained rise in these markers starting on day 20 of stress. Furthermore, our findings extend beyond systemic effects to the liver, a primary regulator of metabolism and redox state. The elevated levels of IL-6 and IL-1β in liver tissue homogenates suggest that CUMS creates a pro-inflammatory microenvironment in the liver, likely due to the organ's role in modulating glucocorticoid-induced metabolic alterations. The literature suggests that chronic stress conditions eventually deplete antioxidant defenses, leading to tissue-specific damage, including lipid peroxidation and protein oxidation (Li et al. 2003).

A significant finding in our study involves the administration of Alamandine, a component of the protective arm of the Renin-Angiotensin System (RAS) that acts through the MrgD receptor. Treatment with Alamandine appeared to modulate the CUMS-induced inflammatory response, particularly by reducing IL-1β in liver tissue and preventing the extreme elevations of serum cytokines. However, the involvement of the Mas receptor in this specific model warrants a nuanced interpretation. The application of the Mas receptor antagonist A779, both alone and in combination with Alamandine, did not consistently reverse the protective effects of Alamandine in a uniform manner. Specifically, the CUMS + Ala + A779 group exhibited significantly higher serum IL-6 levels than the control and Ala-only groups, suggesting that blocking the Mas receptor while providing exogenous Alamandine may exacerbate systemic inflammation. Since A779 targets the Mas receptor rather than the MrgD receptor, these results imply that Alamandine’s anti-inflammatory benefits are likely mediated through the MrgD pathway and that the Mas receptor may provide a separate, perhaps synergistic, level of protection that is lost upon antagonism.

The close relationship between inflammation and oxidative stress represents a fundamental pathophysiological mechanism frequently reported in chronic stress models (Moylan et al. 2014). Increased levels of pro-inflammatory cytokines not only activate the immune response but also stimulate the production of reactive oxygen species (ROS), thereby disrupting cellular redox balance. This process can impair mitochondrial function, weaken antioxidant defense systems, and promote oxidative damage to cellular macromolecules (López-Armada et al. 2013). For this reason, evaluating inflammatory responses together with oxidative stress parameters provides a more comprehensive understanding of tissue-level alterations induced by chronic stress. The evaluation of oxidative stress markers further supports this interpretation. When the activities of the antioxidant enzymes SOD and catalase are evaluated together with MDA, a marker of lipid peroxidation, the findings suggest that chronic stress may influence redox homeostasis in liver tissue. Although MDA levels did not differ significantly among the groups, alterations in SOD and CAT activities suggest a potential adaptive response of the antioxidant defense system to stress-induced oxidative challenges. Such enzymatic changes may reflect compensatory mechanisms aimed at maintaining redox balance under conditions of chronic stress (Lucca et al. 2009; Shen et al. 2024). These observations further support the concept that inflammatory signaling and oxidative stress pathways are closely interconnected processes during prolonged stress exposure (Khansari et al. 2009).

Importantly, the administration of Alamandine appeared to modulate these processes. In our study, Alamandine treatment tended to attenuate the inflammatory and oxidative disturbances induced by CUMS, suggesting a protective role against stress-related redox imbalance. The reduction in lipid peroxidation and the tendency toward normalization of antioxidant enzyme activities indicate that Alamandine may help restore redox homeostasis in liver tissue under chronic stress conditions. However, despite these modulatory effects, serum AST, ALT, and total protein levels remained unchanged across groups, further supporting the notion that the observed alterations primarily reflect molecular and biochemical adaptations rather than overt liver injury.

Histopathological evaluation of both intestinal and colonic tissues provided further insight into the local effects of chronic stress and the potential protective role of Alamandine on intestinal and colonic mucosal morphology. In the control group, both the intestinal and colonic sections exhibited normal mucosal architecture, characterized by intact epithelial layers, organized glandular structures, and a normal distribution of goblet cells. These findings indicate preserved mucosal morphology and absence of inflammatory infiltration under physiological conditions. The intestinal mucosal barrier is primarily composed of epithelial cells, mucus layers, and immune components that together maintain gut homeostasis and protect underlying tissues from luminal pathogens and antigens (Song et al. 2023; Vancamelbeke and Vermeire 2017). Goblet cells play a central role in this barrier by secreting mucins that form a protective mucus layer over the epithelium, thereby preventing microbial invasion and maintaining mucosal integrity (Kim and Ho 2010; Yang and Yu 2021).

In contrast, the CUMS group demonstrated clear histopathological alterations in the small intestine, including villus degeneration and inflammatory cell infiltration within the lamina propria. These findings suggest that chronic unpredictable stress induces alterations in the intestinal mucosa and local inflammatory changes. Previous studies have shown that chronic stress models such as CUMS impair intestinal barrier function by increasing epithelial permeability, altering mucosal morphology, and inducing inflammatory responses in the intestinal mucosa (Kelly et al. 2015). Moreover, stress-induced activation of neuroendocrine and immune pathways can promote recruitment of inflammatory cells within the lamina propria and contribute to mucosal damage (Foster et al. 2017). Interestingly, although morphological deterioration was evident, villus length did not differ significantly among the groups. This suggests that CUMS primarily induced qualitative mucosal damage rather than measurable structural shortening of the villi within the experimental period, as early stress-related intestinal injury may initially manifest as epithelial disruption and inflammatory infiltration before prominent villus atrophy develops (Kelly et al. 2015). The colon showed a somewhat different response pattern. Compared with the pronounced villus alterations observed in the small intestine, colonic tissue displayed relatively milder architectural changes. Still, it exhibited signs of inflammatory involvement and epithelial disturbance in the CUMS group. This difference may reflect the distinct structural and functional characteristics of the colon, which lacks villi and instead contains crypt-based mucosal architecture. Therefore, while chronic stress affected both intestinal segments, the histopathological manifestations were more prominent in the small intestine than in the colon.

A notable common feature in both tissues was the alteration in goblet cell numbers. In the CUMS group, goblet cell density was markedly reduced, suggesting impaired mucus production and compromised mucosal barrier function. Since goblet cells secrete mucins that form the protective mucus layer, their reduction may facilitate inflammation and increase susceptibility to luminal stressors and microbial translocation. Goblet cells play a critical role in maintaining intestinal barrier integrity by producing mucins that form the first protective layer between luminal contents and the epithelium (Johansson et al. 2008; Kim and Ho 2010). Therefore, depletion of goblet cells is closely associated with impaired mucosal defense and increased intestinal inflammation (Birchenough et al. 2015). These findings are consistent with previous reports showing that chronic stress is associated with alterations in intestinal morphology, mucus secretion, and inflammatory responses in the gastrointestinal tract (Foster et al. 2017; Kelly et al. 2015).

Administration of Alamandine appeared to ameliorate many of these stress-induced histological alterations. In the CUMS + Ala group, intestinal villus morphology was better preserved and inflammatory infiltration was reduced compared with the untreated CUMS group. Similarly, colonic tissues exhibited improved epithelial organization and reduced inflammatory changes following Alamandine treatment. These improvements suggest that Alamandine may exert protective effects on stress-induced intestinal histopathological alterations, likely through its anti-inflammatory and regulatory actions associated with the protective arm of the renin–angiotensin system. Growing evidence indicates that components of the alternative RAS axis, including Angiotensin-(1–7) and Alamandine, possess anti-inflammatory and tissue-protective properties that counterbalance the classical Ang II/AT1R pathway and help maintain epithelial homeostasis (Lautner et al. 2013; Santos et al. 2019).

The involvement of the Mas receptor in these effects appeared to be complex. While the CUMS + A779 group continued to show mucosal damage and inflammatory features in both intestinal and colonic tissues, the CUMS + Ala + A779 group demonstrated notable improvement in mucosal architecture, in some aspects approaching that of the control group. Furthermore, goblet cell numbers increased significantly in the treatment groups compared with the CUMS group, indicating a partial restoration of the mucus-secreting epithelial population. This recovery of goblet cells in both intestinal and colonic tissues suggests that Alamandine may help preserve mucosal architecture and mucus-producing epithelial cells under chronic stress. Goblet cells are essential for maintaining the intestinal mucus layer and protecting epithelial surfaces from luminal antigens and microbial invasion, and their restoration is generally associated with improved mucosal defense and epithelial homeostasis (Birchenough et al. 2015; Johansson et al. 2008).

Taken together, these histological findings complement the biochemical and inflammatory data, indicating that chronic unpredictable stress induces mucosal inflammation and epithelial alterations throughout the gut, with more pronounced structural disruption in the small intestine compared to the colon. Chronic stress has been shown to impair intestinal barrier integrity through neuroendocrine and immune mechanisms, leading to increased inflammatory infiltration and epithelial dysfunction. Alamandine administration appears to mitigate these changes and promote restoration of mucosal architecture and goblet cell populations, supporting its potential protective role against chronic stress–induced intestinal and colonic histopathological alterations. These protective effects are consistent with the regulatory functions attributed to the alternative renin–angiotensin system, in which peptides such as Angiotensin-(1–7) and Alamandine exert anti-inflammatory, cytoprotective, and tissue-restorative actions that counterbalance the classical Ang II/AT1R pathway (Lautner et al. 2013; Santos et al. 2018). Although alamandine demonstrated beneficial effects in the present preclinical study, no clinically approved therapeutic dose currently exists, and further pharmacokinetic and safety studies are required before translation to clinical practice.

## Conclusion

In conclusion, our findings demonstrate that chronic unpredictable mild stress induces systemic inflammation, oxidative imbalance, and structural alterations in intestinal tissues without producing overt liver dysfunction, as reflected by the unchanged AST, ALT, and total protein levels. Importantly, Alamandine administration attenuated several of these stress-induced disturbances, including elevations in inflammatory cytokines and oxidative stress parameters, as well as histological damage in intestinal tissues, while partially restoring goblet cell numbers and mucosal integrity. These findings are consistent with the proposed role of the alamandine/MrgD axis in regulating inflammatory and oxidative pathways. However, direct mechanistic confirmation was beyond the scope of the present study. Overall, Alamandine appears to mitigate stress-induced inflammatory and oxidative processes and preserve gut barrier integrity, highlighting its potential as a therapeutic candidate for chronic stress–associated intestinal inflammation and mucosal injury.

These findings suggest that alamandine may represent a promising therapeutic target for mitigating stress-related inflammatory and oxidative disturbances, warranting further investigation in translational and clinical studies.

## Limitations

The present study has several limitations. Although the protective effects of alamandine were demonstrated through biochemical and histopathological analyses, the underlying molecular signaling pathways were not investigated. In addition, intestinal permeability and the expression of key markers of intestinal barrier integrity, including tight junction proteins such as occludin, claudins, and zonula occludens-1 (ZO-1), were not evaluated due to budgetary constraints. Therefore, conclusions regarding intestinal barrier function and gut–brain axis dysregulation should be interpreted with caution. Future studies focusing on intestinal barrier–related proteins, gut permeability, receptor-specific pathways, and downstream signaling mechanisms are needed to elucidate further the protective effects of alamandine under chronic stress conditions.

## Data Availability

The data supporting this study's findings are available from the corresponding author upon reasonable request. All source data for this work (or generated in this study) are available upon reasonable request.
